# Correction: The impact of demand management strategies on parents’ decision-making for out-of-hours primary care: Findings from a survey in the Netherlands

**DOI:** 10.1136/bmjopen-2016-014605corr1

**Published:** 2017-06-26

Giesen M, Keizer E, van de Pol J*, et al.* The impact of demand management strategies on parents’ decision-making for out-of-hours primary care: findings from a survey in The Netherlands. *BMJ Open* 2017;7:e014605. doi: 10.1136/bmjopen-2016-014605

The article has been corrected since it first published. ‘CI’ has been changed to ‘95% CI’ several times throughout the paper and reference 24 has been added.

